# Engagement of Indigenous peoples in commercial tobacco reform strategies: a scoping review protocol

**DOI:** 10.1136/bmjopen-2024-097952

**Published:** 2025-07-03

**Authors:** Andrew Daniel Perusco, Michelle Kennedy, El-Shadan Tautolo, Hershel Clark, Patricia Nez Henderson, Sydney A. Martinez, Priscilla Nez, Andrew Waa, Sierra Wilcox, Penney Upton, Jeyasakthi Venugopal, Raglan Maddox

**Affiliations:** 1 Australian National University, Canberra, Australian Capital Territory, Australia; 2 School of Medicine and Public Health, The University of Newcastle, Callaghan, New South Wales, Australia; 3 Hunter Medical Research Institute, Newcastle, New South Wales, Australia; 4 AUT Pacific Health Research Centre, Auckland University of Technology, Auckland, New Zealand; 5 Black Hills Center for American Indian Health, Rapid City, South Dakota, USA; 6 Department of Biostatistics and Epidemiology, Hudson College of Public Health, University of Oklahoma Health Sciences Center, Oklahoma City, Oklahoma, USA; 7 Eru Pomare Maori Health Research Centre, University of Otago, Wellington, New Zealand; 8 Health Research Institute, University of Canberra, Canberra, ACT, Australia; 9 Yardhura Walani, National Centre for Aboriginal and Torres Strait Islander Wellbeing Research, National Centre for Epidemiology and Population Health, The Australian National University, Canberra, ACT, Australia

**Keywords:** smoking, tobacco control, Indigenous health, public policy, smokefree

## Abstract

**Abstract:**

**Introduction:**

The tobacco and nicotine industry fuels tobacco-related addiction, disease and death. Indigenous peoples experience a disproportionate burden of commercial tobacco-related morbidity and mortality. Over the past two decades, significant progress has been made in reducing smoking prevalence among Indigenous peoples; however, smoking remains a leading contributor to the burden of death and disease. This review will summarise evidence on commercial tobacco resistance and/or eradication strategies, including policy reforms, in relation to Indigenous peoples across Oceania, the Pacific Islands and North America.

**Methods and analysis:**

This review will follow guidelines from the Preferred Reporting Items for Systematic reviews and Meta-Analyses extension for Scoping Reviews and will be conducted in accordance with the Joanna Briggs Institute (JBI) methodology for scoping reviews. This review will consider academic and grey literature published since 1 January 2000. The following electronic databases will be searched for relevant primary research articles and commentaries: PubMed, Scopus, Informit, Web of Science and PsycINFO. Additional searches will be conducted in ProQuest to identify relevant grey literature. Papers will be screened by two reviewers to determine eligibility, followed by full-text data extraction. Findings will be synthesised descriptively for each review question and by region. Studies included in the review will be assessed against criteria for Indigenous engagement in research.

**Ethics and dissemination:**

This protocol was led by Indigenous interests, needs and rights of Indigenous peoples, consistent with the United Nations Declaration on the Rights of Indigenous Peoples (UNDRIP), the WHO’s Framework Convention on Tobacco Control and ethical practice. This review was conceptualised with Indigenous leadership and through engagement, including but not limited to the Indigenous lived experience of the authors (MK, E-ST, HC, PNH, PH, SAM, AW, SW and RM). This review supports the global goal of eradicating commercial tobacco-related harms – reframing commercial tobacco use as a structurally imposed harm sustained by colonial and commercial forces rather than personal choice. Findings from this review will be shared with Indigenous partners and communities who requested this work and will be submitted for peer-reviewed publication.

**Review registration:**

Open Science Framework https://osf.io/wxqcb

**STRENGTHS AND LIMITATIONS OF THIS STUDY**

- This review was requested by Indigenous peoples and communities involved with the Indigenous Circle of the Society for Research on Nicotine and Tobacco and the Tobacco Free programme to inform local, regional and national strategies towards eradicating tobacco and nicotine-related harms.
- Work will be undertaken by a multidisciplinary team of Indigenous and non-Indigenous researchers and guided by an international network of Indigenous researchers.
- Comprehensive academic and grey literature search designed in consultation with a health specialist librarian.
- Included papers will be assessed against best practices in Indigenous research, including governance and leadership considerations.
- This review will be limited to papers published in English.

## Introduction

 Commercial tobacco is a leading cause of disease and premature death. Globally, it was responsible for more than 8 million deaths in 2019^1^ and is a leading risk factor for premature death and disability, placing significant strain on the healthcare system.^2^ People who quit smoking by the age of 40 years can avoid around 90% of the excess mortality risk associated with continued commercial tobacco use.^3 4^

In 2018, commercial tobacco use was the largest contributor to the health burden among Aboriginal and Torres Strait Islander peoples in Australia, responsible for 37% of all deaths in these populations.^5^ Among Māori people in Aotearoa New Zealand, 23% of all deaths between 2013 and 2015 were attributable to smoking .^6^ There has been considerable success in reducing commercial tobacco prevalence among Indigenous peoples; smoking prevalence declined from 54% in 1994 to 43% in 2018–2019 among Aboriginal and Torres Strait Islander adults in Australia^7^, 37% in 2012^8^ to 29% in 2022^9^ among off-reserve First Nations peoples in Canada and from 35% in 2016–2017 to 17% in 2023–2024 among Māori aged ≥15 years in Aotearoa (New Zealand).^10^ Daily commercial tobacco prevalence remains high among Indigenous populations in countries experiencing or that have experienced colonisation, such as Aotearoa, Australia, Canada and the USA,^11^ as well as countries that have experienced colonisation, such as the Pacific Islands Countries and Territories (PICTs). In 2021, up to 20% of deaths in some PICTs were attributable to tobacco use,^12^ with high current smoking rates in the Federated States of Micronesia (62% in 2012) and Tokelau (59% in 2015) based on limited available data.^13^

Racism and colonisation are important direct determinants of the tobacco epidemic. For example, commercial tobacco smoking was systematically entrenched through colonial processes, including payment in rations of tobacco instead of wages and exclusion in the cash economy and education system.^14^ Among Pacific Islander communities living in Aotearoa New Zealand, commercial tobacco was introduced through colonial process and has become embedded within cultural practices of gift-giving, whereby purchasing and gifting of commercial tobacco is widespread, despite high awareness of tobacco-related harms.^15^ For many American Indians, tobacco is sacred and has a special cultural and spiritual significance. Following colonisation, sacred tobacco was prohibited until 1978, whereas the use of commercial tobacco became widespread and entrenched among American Indians.^16^ Commercial tobacco is a human-made, disease-causing agent that has exploited Indigenous peoples and sustained structural inequities and inequalities. It is fundamentally important to acknowledge the differences between commercial and sacred tobacco use to prevent perpetuating colonialism and the erasure of Indigenous ways of knowing, being and doing.^17^

Given the critical role of the ever-evolving tobacco and nicotine industry and the systems that continue to embolden industry interests^18^ in the disproportionate harms and loss experienced by Indigenous peoples,^19^ Indigenous-determined approaches to eradicating commercial tobacco harms are urgently needed. Limitations to the existing array of tobacco control measures, such as slow progress in reducing smoking prevalence and continuing high prevalence among different social groups within societies,^20^ have prompted research and discourse on commercial ‘tobacco endgame’, elimination or eradication strategies. The ‘tobacco endgame’ has been defined as “…initiatives designed to change or permanently eliminate the structural, political, and social dynamics that sustain the tobacco epidemic, so as to achieve, within a specific time, an endpoint for the tobacco epidemic’.^21^ However, there remain inconsistencies in defining goals and optimal approaches towards eradicating commercial tobacco-related death and disease.^22 23^

Indigenous peoples must be involved in determining the needs, design, governance, implementation and evaluation of policies and programmes geared towards eradicating tobacco-related harms to ensure cultural safety and effectiveness. The Māori Affairs Select Committee was a key instigator for the now repealed Smoke-Free Aotearoa 2025,^24^ where the committee completed a public inquiry into the tobacco industry’s promotion of tobacco and its harms for Māori people and made 42 policy recommendations.^25^ However, eradication goals will vary among diverse Indigenous peoples and in different contexts, including careful considerations around sacred tobacco.^26^

Our preliminary searches in Google Scholar and *ANUSuperSearch* (the search engine managed by the Australian National University Library, including 568 multidisciplinary electronic resources and databases) did not identify any current or in-progress scoping or systematic reviews on this topic. We recognised reviews that have summarised available evidence on tobacco control and cessation interventions for Indigenous populations.^11 27 28^ This review focuses on identifying and synthesising evidence on approaches that go beyond individual cessation efforts and population-level interventions, such as prevention and control measures, to examine structural interventions informed by Indigenous worldviews that position industry and state complicity as core drivers of tobacco-related harms.^29^ The latest report of the US Surgeon General, ‘Eliminating Tobacco-Related Disease and Death: Addressing Disparities’ reinforces the vision for a tobacco-free world through a health equity lens that addresses the social, structural and commercial determinants of health.^30^

This review will (1) summarise the literature on commercial tobacco resistance and eradication strategies in relation to Indigenous peoples, (2) identify gaps in the relevance of these strategies to Indigenous peoples and contexts and (3) assess the extent of Indigenous engagement in relevant studies from across North America, Oceania and PICTs. These regions share a common legacy of colonisation with a disproportionately high fatal burden among Indigenous populations attributable to commercial tobacco.^31^

### Review questions

The scoping review will seek to answer the following questions:

1. To what extent has the current praxis and evidence around tobacco resistance and elimination considered Indigenous peoples from Aotearoa, Australia, Canada, USA and PICTs?
  - What is the measured or expected feasibility, effectiveness and impact of the proposed strategies, with consideration for Indigenous self-determination, cultural safety and potential health and equity implications?
  - Were any challenges discussed around strategy implementation, including proposed solutions or recommendations?
  - What contextual information is provided about key factors that may have precipitated the publication (eg, requested by a community, government policy or commitments)?
2. How were tobacco resistance, elimination, eradication and related strategies defined in Indigenous contexts?
  - Were these definitions developed by Indigenous peoples and/or through meaningful engagement with Indigenous peoples?
3. Does the literature document the engagement of Indigenous peoples in the governance, conception, development, conduct and/or authorship of the identified papers?
  - Which Indigenous peoples and/or communities were involved and at which stages of the research or initiative?

## Methods and Analysis

The proposed scoping review will be conducted in accordance with the Joanna Briggs Institute (JBI) methodology^32^ and will be reported as per the Preferred Reporting Items for Systematic Reviews and Meta-Analyses (PRISMA) extension for Scoping Reviews Checklist (see online supplemental appendix I).^33^ In the development of this protocol, we consulted with the *Indigenous Circle of the Society for Research on Nicotine and Tobacco (SRNT*).

### Inclusion criteria

#### Population/context

The scoping review will include papers that considered Indigenous peoples with no age restriction from Aotearoa, Australia, Canada, USA and PICTs in relation to tobacco resistance and eradication strategies.

#### Concept

Papers will be included if they examined tobacco resistance, elimination and/or eradication strategies, goals and visions. Table 1 provides an outline of possible tobacco endgame or eradication proposals based on a review by McDaniel, Smith and Malone.^22^ Papers with an exclusive focus on individual-level interventions (eg, cessation support) with no evidence on broader strategies geared towards the eradication of commercial tobacco harms will be excluded.

**Table 1:** Examples of tobacco endgame or eradication proposals based on a review by McDaniel, Smith and Malone (2016)^22^

Focus of endgame proposals	Examples
Product	Regulate nicotine levels to make cigarettes non-addictive or less addictive Redesign the cigarette to make it unappealing E-cigarettes (in the context of making cigarettes less appealing)
User	Smoker’s licence Prescription to purchase tobacco Restrict sales to year born
Market/supply	Licensing, outlet restrictions, display bans and price controls Ban combustibles Regulate to advantage cleaner nicotine products over combustibles Quota/sinking lid (quota on manufacture and imports): ‘As quotas were reduced, prices for the shares and consequently prices for tobacco products would rise, until demand shrank’. Price caps to reduce tobacco company profitability.
Institutional structure	Tobacco control agency: ‘Such an agency would manage products, marketing, development of less harmful/addictive products, price, sales and monitoring of the regulatory system’. Regulated market model: ‘This agency could set standards for manufacturers (from whom it would buy) as well as for retailers (to whom it would supply products). This system could permit innovation (eg, the agency would buy demonstrably safer products) while controlling price, packaging and promotion’. State takeover of tobacco companies: ‘…suggests that tobacco companies be purchased and managed with a health promotion mandate, which could then use multiple strategies to meet mandated tobacco use reduction goals’. Performance-based regulation: ‘…a public agency set goals for reductions in smoking prevalence that tobacco companies would be.’ required to meet within a certain time frame and measure whether those goals were met”. Integrated endgame strategies, such as combinations of the proposals mentioned in this table.

In line with ethical principles of conducting research with Indigenous peoples,^34 35^ this review will also identify and describe the extent to which Indigenous peoples have led or been actively involved in the governance, conception, development, conduct and/or reporting of the identified papers. This will be achieved by assessing Indigenous engagement against eight research reporting domains (CONSIDER checklist)^34^ and the SRNT – Oceania’s ethical principles for research with Indigenous peoples.^35^ Table 2 provides a summary of the eligibility criteria for this review, along with possible reasons for the exclusion of papers identified from the search.

**Table 2:** Eligibility criteria for scoping review

Inclusion criteria	Possible reasons for exclusion
*Population:*Included Indigenous peoples from one or more of the following regions: Aotearoa, Australia, Canada, USA and Pacific Islands Countries and Territories. No age restrictions.	Broad statements on implications/considerations for Indigenous peoples around commercial tobacco resistance with no reported engagement of Indigenous peoples. Inclusion of data on Indigenous peoples with no reported engagement of Indigenous peoples. Based on the CONSIDER checklist’s domain 3 on research relationships, research concerning Indigenous peoples should abide by Indigenous ethical guidelines, including meaningful engagement, and involvement and partnership of Indigenous peoples and communities in research processes (eg, through participatory research).
*Intervention:*Examined commercial tobacco resistance, elimination and/or eradication strategies, goals, and visions such as those summarised in table 1.	Exclusive focus on individual-level interventions, such as smoking cessation. Exclusive focus on ceremonial or traditional use of tobacco.
*Comparator:*No specific comparator groups are required for inclusion.	None.
*Outcomes:*Authors reported engagement of Indigenous peoples, including the extent of engagement (when engaged, type of involvement, etc.). This outcome will be assessed against the CONSIDER checklist’s eight research domains.Other outcomes of interest but not required for inclusion: either a qualitative and/or quantitative assessment of feasibility, acceptability, effectiveness and cultural safety.	No mention of engagement with Indigenous peoples.

#### Types of evidence sources

This scoping review will include journal articles published in peer-reviewed journals and academic theses. Primary papers, including experimental, quasi-experimental, observational, ‘natural experiments’, qualitative studies, mixed methods and modelling studies will be included. Standalone commentaries and analyses (including theoretical analyses or public policy discourse) will be included. Reviews will be excluded, but references in relevant reviews will be considered for inclusion.

### Search strategy

PubMed, Scopus, Informit Indigenous Collection, Web of Science and PsycINFO will be searched to identify relevant indexed academic literature. Relevant grey literature documents, including dissertations and government reports, will be identified in ProQuest. Additional searches may be conducted through consultation with the Indigenous Circle of SRNT.

An initial search in PubMed was conducted to identify keywords from relevant articles. A comprehensive list of search terms for specific Indigenous populations of interest was adapted from a systematic review on effective knowledge translation in Indigenous health research.^36^ A full search strategy for this protocol was piloted in Scopus on 6 December 2024 (online supplemental appendix II). The search strategy and keywords will be adapted for each information source as required. Non-English language papers will be excluded, as translation services are beyond the resources available to this project. An Australian National University librarian assisted in the development of the search strategy.

As Aotearoa New Zealand was the earliest jurisdiction to consider tobacco elimination in 2010, searches will be limited to papers published from 1 January 2000 onwards to encapsulate work leading up to Aotearoa New Zealand’s SmokeFree 2025 action plan.

### Study selection

Search results from each information source will be collated and imported into EndNote 21 (Clarivate Analytics, PA, USA). De-duplicated references will then be exported into Covidence to support title/abstract screening and full-text data extraction. The process for inclusion by title/abstract will first be piloted independently by three reviewers using 25 randomly selected papers. The title/abstract of the papers will then be reviewed against the inclusion criteria by two reviewers. The articles selected for potential inclusion will then undergo full-text review by two reviewers, and reasons for exclusion at the full-text stage will be documented and reported in the final review. Disagreements between reviewers at any stage of the review will be resolved through consensus or in consultation with a third author. Initial search yields up until final inclusion will be detailed in full in the final scoping review and presented in a PRISMA flow diagram.^37^

### Data extraction

A draft data extraction tool has been developed for this review (online supplemental appendix III). This tool will be piloted by two reviewers to ensure that relevant information is adequately captured and refined as necessary. Any modifications to the tool will be detailed in the final scoping review. Through this tool, specific information will be collected from eligible articles regarding proposed tobacco resistance, elimination and eradication strategies, any specific populations covered or considered by the strategies and the extent of Indigenous engagement. Additional information will be collected on any narrative or quantitative (including modelling) assessments or evaluations of the proposed strategies regarding effectiveness and effect (eg, cultural safety and acceptability). We will also identify and code examples of tobacco and nicotine industry framing, such as ‘personal responsibility’, ‘freedom of choice’ or ‘harm reduction’, which obscure structural and commercial determinants of health and well-being.^38^ Any discrepancies in the information extracted from papers between the two reviewers will be resolved through full-text review and dialogue within the review team, including an Indigenous researcher(s) as required, in alignment with Indigenous data sovereignty principles.

### Data analysis and presentation

Data extracted from the included papers will be summarised and discussed in the context of the review questions and by region. As colonisation is also a determinant of the tobacco epidemic, a de-colonising lens will be used in appraising individual papers, specifically by assessing each paper against the eight research domains for reporting of health research involving Indigenous peoples (CONSIDER checklist)^34^: governance, relationships, prioritisation, methodologies, participation, capacity, analysis and findings and dissemination. In addition to CONSIDER, the review will apply a framing analysis guided by the Resisting Industry Narratives framework to help identify industry language and deflection tactics that shift accountability away from the tobacco industry.^38^

To identify priorities for research, gaps will be discussed as they relate to current policy discourse for Indigenous tobacco resistance, elimination or eradication.

### Expected outcomes

The proposed scoping review will summarise published information on visions, goals and approaches to commercial tobacco eradication that supports the health, well-being and aspirations of Indigenous peoples, including any identified strengths, challenges and evaluations of proposed approaches. Findings may help contextualise the needs and priorities of Indigenous peoples as it relates to commercial tobacco, as well as necessary conditions and policies that would support the assertion of Indigenous sovereignty in implementing actions to prevent further harms. Importantly, this review is expected to help shift the field toward structural and system-level reform(s) rather than individualised health approaches. Findings will also directly support the work of the Tackling Indigenous Smoking (TIS) program across Australia, funded by the Australian Government Department of Health, Disability and Ageing. The overarching goal of the TIS program is to improve the health and well-being of Aboriginal and Torres Strait Islander peoples through community-led, locally tailored actions geared towards eliminating and/or eradicating commercial tobacco and vaping-related harms. This review will contribute to the development of Indigenous-led abolitionist strategies that aim to dismantle the structural conditions enabling the commercial tobacco epidemic.^29 38^

### Ethics and dissemination

This review will involve collecting and summarising published information that matches the proposed scope of this work; no primary data will be collected. As such, a formal ethics committee review was not sought. Findings from this review will be shared with Indigenous partners and communities who requested this work and will be submitted for peer-reviewed publication. Additionally, authors may present this review at national public health conferences to broaden the influence of this work.

### Patients and public involvement

This protocol was conceived and informed by the expressed needs of Indigenous peoples and communities, including but not limited to the Indigenous lived experience of the majority of authors (MK, E-ST, HC, PNH, PH, SAM, PN, AW, SW and RM) and an extended international network of Indigenous researchers (Indigenous Circle of SRNT). The work for this review will continue to be directed by Indigenous peoples and communities throughout the life of the project, including dissemination.

## Supplementary material

10.1136/bmjopen-2024-097952 online supplemental material 1

## References

[1] Murray CJL, Aravkin AY, Zheng P (2020). Global burden of 87 risk factors in 204 countries and territories, 1990–2019: a systematic analysis for the Global Burden of Disease Study 2019. The Lancet.

[2] Institute for Health Metrics and Evaluation (2018). Findings from the global burden of disease study 2017.

[3] Jha P, Ramasundarahettige C, Landsman V (2013). 21st-century hazards of smoking and benefits of cessation in the United States. N Engl J Med.

[4] Banks E, Joshy G, Weber MF (2015). Tobacco smoking and all-cause mortality in a large Australian cohort study: findings from a mature epidemic with current low smoking prevalence. BMC Med.

[5] Thurber KA, Banks E, Joshy G (2021). Tobacco smoking and mortality among Aboriginal and Torres Strait Islander adults in Australia. Int J Epidemiol.

[6] Walsh M, Wright K (2020). Ethnic inequities in life expectancy attributable to smoking. NZ Med J.

[7] Thurber KA, Walker J, Maddox R (2020). A review of evidence on the prevalence of and trends in cigarette and e-cigarette use by Aboriginal and Torres Strait Islander youth and adults.

[8] Statistics Canada (2024). Smoking status by Aboriginal identity.

[9] Statistics Canada (2024). Smoking status of First Nations people living off reserve, Métis and Inuit, by age group and gender. https://www150.statcan.gc.ca/t1/tbl1/en/tv.action?pid=4110007101.

[10] Ministry of Health (2024). Annual data explorer 2023/24: New Zealand health survey [indicator: current smokers (has smoked more than 100 cigarettes in lifetime and currently smokes at least once a month)]. https://minhealthnz.shinyapps.io/nz-health-survey-2023-24-annual-data-explorer.

[11] Carson K, Jayasinghe H, Smith B (2024). Smoking cessation and tobacco prevention in Indigenous populations. EB.

[12] Institute for Health Metrics and Evaluation (IHME) (2021). GBD results seattle. https://vizhub.healthdata.org/gbd-results.

[13] Gifford H, Tautolo E-S, McCool JP (2022). Getting there together: highlights, challenges and opportunities for tobacco control in the Oceania region. Tob Control.

[14] Blyton G (2010). Smoking Kills: the introduction of tobacco smoking into Aboriginal society with a particular focus on the Hunter Region of Central Eastern New South Wales from 1800 to 1850. Int J Crit Indig Stud.

[15] Tautolo E-S, Edwards R, Gifford H (2015). A gift and a burden: the purchase and distribution of duty-free tobacco and its potential impact upon Pacific people in New Zealand. Tob Control.

[16] D’Silva J, O’Gara E, Villaluz NT (2018). Tobacco industry misappropriation of American Indian culture and traditional tobacco. Tob Control.

[17] Jetty R (2017). Canadian Paediatric Society FN, Inuit, Métis Health Committee O, Ontario. Tobacco use and misuse among Indigenous children and youth in Canada. Paediatr Child Health.

[18] Waa A, Maddox R, Nez Henderson P (2020). Big tobacco using Trojan horse tactics to exploit Indigenous peoples. Tob Control.

[19] Australian Institute of Health Welfare (2024). Australian institute of health welfare. size and sources of the health gap for australia’s first 49 nations people 2017–2019.

[20] Gartner C (2020). CREATE a new path to a smoke-free Australia Sydney: Australian medical association. https://insightplus.mja.com.au/2020/40/create-a-new-path-to-a-smoke-free-australia.

[21] Malone R, McDaniel P, Smith E (2014). It is time to plan the tobacco endgame. BMJ.

[22] McDaniel PA, Smith EA, Malone RE (2016). The tobacco endgame: a qualitative review and synthesis. Tob Control.

[23] Novotny TE (2015). The tobacco endgame: is it possible?. PLoS Med.

[24] Ball J, Stanley J, Wilson N (2016). Smoking prevalence in New Zealand from 1996–2015: a critical review of national data sources to inform progress toward the smokefree 2025 goal. NZ Med J.

[25] New Zealand Parliament (2018). Briefing on achieving the Smokefree 2025 goal for New Zealand [Kaupapa whakamōhio ki te ekenga o te whāinga Auahi Kore 2025 mō Aotearoa]: Report of the Health Committee and the Māori Affairs Committee.

[26] Henderson PN, Lee JP, Soto C (2021). Decolonization of Tobacco in Indigenous Communities of Turtle Island (North America). Nicotine Tob Res.

[27] Minichiello A, Lefkowitz ARF, Firestone M (2016). Effective strategies to reduce commercial tobacco use in Indigenous communities globally: A systematic review. BMC Public Health.

[28] Chamberlain C, Perlen S, Brennan S (2017). Evidence for a comprehensive approach to Aboriginal tobacco control to maintain the decline in smoking: an overview of reviews among Indigenous peoples. Syst Rev.

[29] Maddox R, Telford RM, Waa A (2024). Eradication of commercial tobacco related disease and death. Tob Control.

[30] U.S. Department of Health and Human Services (2024). Eliminating tobacco-related disease and death: addressing disparities - a report of the surgeon general. U.S. Department of Health and Human Services, Centers for Disease Control and Prevention, National Center for Chronic Disease Prevention and Health Promotion, Office on Smoking and Health.

[31] Stevenson L, Campbell S, Bohanna I (2017). Establishing Smoke-Free Homes in the Indigenous Populations of Australia, New Zealand, Canada and the United States: A Systematic Literature Review. Int J Environ Res Public Health.

[32] Peters M, Godfrey C, McInerney P, Aromataris E, Munn Z (2020).

[33] Tricco AC, Lillie E, Zarin W (2018). PRISMA Extension for Scoping Reviews (PRISMA-ScR): Checklist and Explanation. Ann Intern Med.

[34] Huria T, Palmer SC, Pitama S (2019). Consolidated criteria for strengthening reporting of health research involving indigenous peoples: the CONSIDER statement. BMC Med Res Methodol.

[35] Society for Research on Nicotine and Tobacco-Oceania (2021). SRNT-oceania statement on 40 acknowledgement statement and ethical principles for research with Indigenous peoples. https://docs.google.com/document/d/1L_5kE-csK2jZfk2u6lTwF2z1lHsYLaCD/edit.

[36] Morton Ninomiya ME, Atkinson D, Brascoupé S (2017). Effective knowledge translation approaches and practices in Indigenous health research: a systematic review protocol. Syst Rev.

[37] Page MJ, McKenzie JE, Bossuyt PM (2021). The PRISMA 2020 statement: an updated guideline for reporting systematic reviews. BMJ.

[38] Maddox R, Diaz A, Waa A (2024). Resisting industry narratives: guidance to avoid tobacco and nicotine industry framing. Health Promot Int.

