# Supplementary material for: Engagement of Indigenous peoples in commercial tobacco reform strategies: a scoping review protocol
Source: BMJ Open. 2025 Jul 3;15(7):e097952. doi: 10.1136/bmjopen-2024-097952 (PMC12226939; doi:10.1136/bmjopen-2024-097952)
Supplement: online supplemental material 1 [file bmjopen-15-7-s001.docx]

# SUPPLEMENTARY FILE

**Appendix I: Completed Preferred Reporting Items for Systematic reviews and Meta-Analyses extension for Scoping Reviews (PRISMA-ScR) checklist**

| **SECTION** | **ITEM** | **PRISMA-ScR CHECKLIST ITEM** | **REPORTED ON PAGE #** |
| --- | --- | --- | --- |
| **TITLE** | | | |
| Title | 1 | Identify the report as a scoping review. | Page 1, Lines 1-2 |
| **ABSTRACT** | | | |
| Structured summary | 2 | Provide a structured summary that includes (as applicable): background, objectives, eligibility criteria, sources of evidence, charting methods, results, and conclusions that relate to the review questions and objectives. | Page 1, Lines 4-31 |
| **INTRODUCTION** | | | |
| Rationale | 3 | Describe the rationale for the review in the context of what is already known. Explain why the review questions/objectives lend themselves to a scoping review approach. | Page 3, Lines 33-45; Page 4, Line 1 |
| Objectives | 4 | Provide an explicit statement of the questions and objectives being addressed with reference to their key elements (e.g., population or participants, concepts, and context) or other relevant key elements used to conceptualize the review questions and/or objectives. | Page 4, Lines 17-20 |
| **METHODS** | | | |
| Protocol and registration | 5 | Indicate whether a review protocol exists; state if and where it can be accessed (e.g., a Web address); and if available, provide registration information, including the registration number. | Page 1, Line 31 |
| Eligibility criteria | 6 | Specify characteristics of the sources of evidence used as eligibility criteria (e.g., years considered, language, and publication status), and provide a rationale. | Page 6, Table 2 |
| Information sources* | 7 | Describe all information sources in the search (e.g., databases with dates of coverage and contact with authors to identify additional sources), as well as the date the most recent search was executed. | Page 7, Lines 9-23 |
| Search | 8 | Present the full electronic search strategy for at least 1 database, including any limits used, such that it could be repeated. | Supplementary File, Appendix II |
| Selection of sources of evidence† | 9 | State the process for selecting sources of evidence (i.e., screening and eligibility) included in the scoping review. | Page 7, 25-34 |
| Data charting process‡ | 10 | Describe the methods of charting data from the included sources of evidence (e.g., calibrated forms or forms that have been tested by the team before their use, and whether data charting was done independently or in duplicate) and any processes for obtaining and confirming data from investigators. | Page 7, Lines 36-41; Page 8, Lines 1-8 |
| Data items | 11 | List and define all variables for which data were sought and any assumptions and simplifications made. | Supplementary File, Appendix III |
| Critical appraisal of individual sources of evidence§ | 12 | If done, provide a rationale for conducting a critical appraisal of included sources of evidence; describe the methods used and how this information was used in any data synthesis (if appropriate). | None. |
| Synthesis of results | 13 | Describe the methods of handling and summarizing the data that were charted. | Page 8, Lines 10-19 |
| **RESULTS** | | | |
| Selection of sources of evidence | 14 | Give numbers of sources of evidence screened, assessed for eligibility, and included in the review, with reasons for exclusions at each stage, ideally using a flow diagram. | Not applicable (protocol) |
| Characteristics of sources of evidence | 15 | For each source of evidence, present characteristics for which data were charted and provide the citations. | Not applicable (protocol) |
| Critical appraisal within sources of evidence | 16 | If done, present data on critical appraisal of included sources of evidence (see item 12). | Not applicable (protocol) |
| Results of individual sources of evidence | 17 | For each included source of evidence, present the relevant data that were charted that relate to the review questions and objectives. | Not applicable (protocol) |
| Synthesis of results | 18 | Summarize and/or present the charting results as they relate to the review questions and objectives. | Not applicable (protocol) |
| **DISCUSSION** | | | |
| Summary of evidence | 19 | Summarize the main results (including an overview of concepts, themes, and types of evidence available), link to the review questions and objectives, and consider the relevance to key groups. | Not applicable (protocol) |
| Limitations | 20 | Discuss the limitations of the scoping review process. | Page 2, Line 1 |
| Conclusions | 21 | Provide a general interpretation of the results with respect to the review questions and objectives, as well as potential implications and/or next steps. | Page 8, Lines 21-34 |
| **FUNDING** | | | |
| Funding | 22 | Describe sources of funding for the included sources of evidence, as well as sources of funding for the scoping review. Describe the role of the funders of the scoping review. | Page 9, Lines 6-10 |

JBI = Joanna Briggs Institute; PRISMA-ScR = Preferred Reporting Items for Systematic reviews and Meta-Analyses extension for Scoping Reviews.

* Where *sources of evidence* (see second footnote) are compiled from, such as bibliographic databases, social media platforms, and Web sites.

† A more inclusive/heterogeneous term used to account for the different types of evidence or data sources (e.g., quantitative and/or qualitative research, expert opinion, and policy documents) that may be eligible in a scoping review as opposed to only studies. This is not to be confused with *information sources* (see first footnote).

‡ The frameworks by Arksey and O’Malley (6) and Levac and colleagues (7) and the JBI guidance (4, 5) refer to the process of data extraction in a scoping review as data charting*.*

§ The process of systematically examining research evidence to assess its validity, results, and relevance before using it to inform a decision. This term is used for items 12 and 19 instead of "risk of bias" (which is more applicable to systematic reviews of interventions) to include and acknowledge the various sources of evidence that may be used in a scoping review (e.g., quantitative and/or qualitative research, expert opinion, and policy document).

*From:* Tricco AC, Lillie E, Zarin W, O'Brien KK, Colquhoun H, Levac D, et al. PRISMA Extension for Scoping Reviews (PRISMAScR): Checklist and Explanation. Ann Intern Med. 2018;169:467–473. [doi: 10.7326/M18-0850](http://annals.org/aim/fullarticle/2700389/prisma-extension-scoping-reviews-prisma-scr-checklist-explanation).

## Appendix II: Search strategy

The following search was completed on December 6, 2024 in the Scopus search engine.

| **Search** | **Query** | **Records retrieved** |
| --- | --- | --- |
| #1 | TITLE-ABS-KEY (cigar* OR tobacco OR smok* OR nicotin*) | **1,075,044** |
| AND #2 | TITLE-ABS-KEY (endgame OR "end game" OR eliminat* OR eradicat* OR prohibit* OR "phas*out" OR "phas* out" OR aboli* OR smokefree OR "smoke free" OR "nicotine content" OR "very low nicotine" OR VLNC OR "nicotine reduction" OR "reduced nicotine" OR denicotinis* OR "nicotine level" OR "maximum nicotine" OR "product standard*" OR content OR ingredient OR constituent OR flavour* OR flavor* OR additive* OR menthol* OR toxicant* OR emission* OR pH OR filter OR "alternative nicotine" OR "less harmful nicotine" OR "e-cigarette" OR "electronic cigarette" OR vaping OR vape OR "electronic nicotine" OR vaporiser* OR vaporizer* OR snus OR juul OR snuff OR "non-smoked" OR "low nitrosamine" OR smokeless OR "reduced exposure product" OR "nicotine substitute" OR "user's license" OR "smoker's license" OR "user's licence" OR "smoker's licence" OR licens* OR licenc* OR "tobacco on prescription" OR "cigarettes on prescription" OR "prescription before purchasing tobacco" OR "prescription to purchase tobacco" OR "prescribe cigarettes" OR "prescription-only tobacco" OR "cigarette* prescription only" OR "tobacco prescription only" OR "tobacco free generation" OR "smoke free generation" OR "age of sale" OR "sinking lid" OR quota OR "corporate manslaughter" OR compensation OR litigation OR profitability OR "industry viability" OR "viability of the tobacco industry" OR "regulated market model" OR monopson* OR "non-profit agency" OR "tobacco control agency" OR "tobacco use management" OR "sale* ban" OR "cig* ban*" OR "less attract*" OR "reduc* attract*" OR "less addict*" OR "reduc* addict*" OR "eliminat* disparit*" OR "eliminat* inequit*" OR "outlet* density" OR "outlet* restrict*" OR "outlet* reduc*" OR "end* the tobacco epidemic" OR "end* the epidemic" OR "pharm* only" OR "only pharm*") | **11,218,496**  **#1 AND #2=169,681** |
| AND #3 | TITLE-ABS-KEY (Aborigin* OR Indigenous OR Eskimo* OR Inuit* OR Inuk* OR Metis OR "First Nation*" OR "1st nation*" OR "Native Canadian*" OR "Native American*" OR Maori* OR Māori* OR "Pacific Islander*" OR "American Indian*" OR Amerindian* OR "Native Alaska*" OR "Alaska Native*" OR "Native Hawaiian*" OR "Torres Strait Islander*" OR "on-reserve" OR "off-reserve" OR tribal OR autochtone* OR amerindien* OR indigene*) | **309,800** |
| OR #4 | TITLE-ABS-KEY ("american native continental ancestry group" OR "north American indians" OR inuits OR "Oceanic Ancestry Group" OR "United States Indian Health Service" OR "indigenous Health Services") | **13,911** |
| OR #5 | TITLE-ABS-KEY (Cree OR Inuktitut OR Ojibway OR Innu OR Dene OR "Oji-Cree" OR Anishinini OR Mikmaq OR Dakota OR Sioux OR Atikamekw OR Blackfoot OR Inuit OR Metis OR Nuxalk) | **31,089** |
| OR #6 | TITLE-ABS-KEY ("Alaska Athabascan" OR Ahtna OR Deg Hitan OR Denaina OR Gwichin OR Han OR Holikachuk OR Kolchan OR Koyukon OR "Lower Tanana" OR Tanacross OR "Upper Tanana" OR Eyak OR Haida OR Tlingit OR Tsimshian OR Eskimo OR Inupiat OR Yupik OR Cupik OR Sugpiaq OR Alutiiq OR Alutiqu OR Chugach OR Koniag OR Aleut OR Unangan) | **71** |
| OR #7 | TITLE-ABS-KEY (Bannock OR “Coso People” OR Kawaiisu OR Mono OR Timbisha OR Panamint OR Koso OR Washo OR Palagewan OR Pahkanapil OR Kucadikadi OR “Owens Valley Paiute” OR Chemehuevi OR Kaibab OR Kaiparowtis OR Moapa OR Panaca OR Panguitch OR Paranigets OR Shivwits OR Guchundeka OR Kuccuntikka OR Tukkutikka OR Tukudeka OR Bohoinee OR Pohoini OR Pohogwe OR “Sage Grass people” OR “Sagebrush Butte People” OR Agaideka OR Doyahinee OR Kammedeka OR Kammitikka OR Hukunduka OR Tukudeka OR Dukundeka OR Yahandeka OR Yakandika OR Kusiutta OR Goshute OR Gosiute OR Kuyatikka OR Kuyudikka OR Mahaguaduka OR Painkwitikka OR Pasiatikka OR Tipatikka OR Tsaiduka OR Tsogwiyuyugi OR Waitikka OR Watatikka OR Wiyimpihtikka OR Capote OR Moanunts OR Muache OR Pahvant OR Sanpits OR Timpanogots OR Uintah OR Uncompahgre OR Taviwach OR Weeminuche OR "White River Utes" OR Parusanuch OR Yampa) | **177,888** |
| OR #8 | TITLE-ABS-KEY (Anishinaabe OR Anishinape OR Anicinape OR eshnabe OR Nishnaabe OR Saulteaux OR Nakawe OR Apache OR Lipan OR "Kiowa Apache" OR Arapaho OR Arapahoe OR Besawunena OR Nawathinehena OR Arikara OR Arikaree OR Arikari OR Atsina OR "Gros Ventre" OR Blackfoot OR "Kainai Nation" OR "Northern Peigan" OR Aapatohsipikani OR Blackfeet OR Siksika OR Cheyenne OR Suhtai OR Comanche OR "Plains Cree" OR Absaroka OR Apsaalooke OR Escanjaques OR Hidatsa OR Ioway OR Kansa OR Kanza OR Kiowa OR Mandan OR Missouria OR Omaha OR Osage OR Otoe OR Oto OR Pawnee OR Chaui OR Kitkehakhi OR Pitahawirata OR Skidi OR Ponca OR Quapaw OR Sioux OR Santee OR Yankton OR Yanktonai OR Lakota OR Teton OR Sichangu OR Brule OR Oglala OR Itazipcho OR Hunkpapha OR Hunkpapa OR Mnikhowozu OR Miniconjou OR Sihasapa OR Oohenunpa OR Nakoda OR Nakota OR Teyas OR Tonkawa OR "Tsuu Tina" OR Wichita OR Kichai OR Rayados OR Taovayas OR Tawakoni OR Waco) | **59,528** |
| OR #9 | TITLE-ABS-KEY (Abenaki OR Tarrantine OR Kennebec OR Caniba OR Anishinaabe OR Anishinape OR Anicinape OR Neshnabe OR Nishnaabe OR Algonquin OR Nipissing OR Ojibwa OR Saulteaux OR Nakawe OR "Odawa people" OR Potawatomi OR Assateague OR Attawandaron OR Beothuk OR "Choptank people" OR Conoy OR Erie OR Etchemin OR Meskwaki OR "Ho-Chunk" OR Winnebago OR Honniasont OR Illiniwek OR Cahokia OR Kaskaskia OR Miami OR Mitchigamea OR Moingona OR Peoria OR Tamaroa OR Wea OR Haudenosaunee OR Cayuga OR Mohawk OR Oneida OR Onondaga OR Seneca OR Mingo OR Tuscarora OR Kickapoo OR Laurentian OR "Lenni-Lenape" OR "Munsee Esopus" OR Waoranecks OR Warranawankongs OR Minisink OR "Ramapough Mountain Indians" OR Unami OR Acquackanonk OR Hackensack OR Navasink OR Raritan OR Rumachenanck OR Haverstraw OR Tappan OR Unalachtigo OR Wiechquaeskecks OR Mascouten OR Massachusett OR Ponkapoag OR Menominee OR Mahican OR Housatonic OR Mahican OR Wappani OR Wappinger OR "Wappinger proper" OR Hammonasset OR Kitchawank OR Kichtawanks OR Kichtawank OR Mattabesset OR Massaco OR Menunkatuck OR Nochpeem OR Paugusset OR Podunk OR Poquonock OR Quinnipiac OR Eansketambawg OR Rechgawawanc OR Recgawawanc OR Sicaog OR Sintsink OR Siwanoy OR Tankiteke OR Tunxis OR Wecquaesgeek OR Wyachtonok OR Massachusett OR Mikmaq OR Micmac OR Mohegan OR Montaukett OR Montauk OR Nanticoke OR Narragansett OR Niantic OR Nipmuc OR Nipmuck OR Occaneechee OR Passamaquoddy OR Patuxent OR Penobscot OR Pequot OR Petun OR Tionontate OR Pocumtuc OR Poospatuck OR Quapaw OR Quinnipiac OR Hammonasset OR Mattabesec OR Mattatuck OR Menunkatuck OR Meriden OR Mioonkhtuck OR Naugatuck OR Nehantic OR Paugusset OR Podunk OR Potatuck OR Totoket OR Tunxis OR Wangunk OR Wepawaug OR Sauk OR Shawnee OR Shinnecock OR Susquehannock OR Tauxenent OR Doeg OR Unquachog OR Wampanoag OR Nauset OR Patuxet OR Pokanoket OR Wawenoc OR Wenro OR Wenrohronon OR Wicocomico OR Wolastoqiyik OR Wyandot) | **39,472** |
| OR #10 | TITLE-ABS-KEY ("Chinook peoples" OR Cathlamet OR Clackamas OR Clatsop OR Kathlamet OR Multnomah OR "Wasco-Wishram" OR Watlata OR "Interior Salish" OR Chelan OR "Coeur dAlene Tribe" OR Entiat OR Flathead OR Selisch OR Salish OR Kalispel OR "Pend dOreilles" OR Methow OR Nespelem OR Nlakapamux OR "Thompson people" OR "Nicola people" OR Okanagan OR Secwepemc OR "Shuswap people" OR Sinixt OR "Sinkiuse-Columbia" OR "Spokane people" OR Statimc OR Lilwat OR "In-SHUCK-ch" OR Wenatchi OR Wenatchee OR Sanpoil OR Sinkayuse OR "Sahaptin people" OR "Upper Cowlitz" OR Taidnapam OR Kittitas OR "Upper Yakima" OR "Klickitat Tribe" OR "Nez Perce" OR Pshwanwapam OR Pswanwapam OR Skinpah OR Tenino OR Warmsprings OR Tygh OR "Upper Deschutes" OR Umatilla OR "Walla Walla" OR Wanapum OR Wauyukma OR Wyam OR "Lower Deschutes" OR Yakama OR Cayuse OR Celilo OR Wayampam OR Cowlitz OR "Fort Klamath" OR Kalapuya OR "northwest Atfalati" OR Tualatin OR "Mohawk River" OR Santiam OR Yaquina OR Kutenai OR Kootenai OR Ktunaxa OR "Lower Snake people" OR Chamnapam OR Wauyukma OR Naxiyampam OR Modoc OR Molala OR Molale OR Palus OR Palouse OR "Upper Nisqually" OR Mishalpan) | **6,230** |
| OR #11 | TITLE-ABS-KEY (Tlingit OR Nisgaa OR Tsetsaut OR Haida OR Tsimshian OR Gitxsan OR Haisla OR Heiltsuk OR Wuikinuxv OR Kwakwakawakw OR "Nuu-chah-nulth" OR Makah OR "Coast Salish" OR Nuxalk OR Willapa OR Chimakum OR Quileute OR Chinook) | **7,297** |
| OR #12 | TITLE-ABS-KEY (Abihka OR Acolapissa OR Colapissa OR Ais OR Alabama OR Alafay OR "Alafia tribe" OR Pojoy OR Pohoy OR Costas OR Alafeyes OR Alafaya Costas OR Amacano OR Apalachee OR Apalachicola OR Atakapa OR Akokisa OR Bidai OR Deadose OR Orcoquiza OR Patiri OR Tlacopsel OR Avoyel OR "Backhooks Nation" OR Chuaque OR Holpaos OR Huaq OR Nuaq OR Pahoc OR Pahor OR Paor OR Uca OR Bayogoula OR Biloxi OR "Boca Ratones" OR "Caddo Confederacy" OR Adai OR Adaizan OR Adaizi OR Adaise OR Adahi OR Adaes OR Adees OR Atayos OR Cahinnio OR Doustioni OR Eyeish OR Hais OR Hainai OR Hasinai OR Kadohadacho OR Nabedache OR Nabiti OR Nacogdoche OR Nacono OR Nadaco OR Nanatsoho OR Nasoni OR Natchitoches OR Neche OR Nechaui OR Ouachita OR Tula OR Yatasi OR Calusa OR "Cape Fear Indians" OR Catawba OR Esaw OR Usheree OR Ushery OR Yssa OR Chacato OR Chakchiuma OR Chatot OR Chacato OR Chactoo OR Chawasha OR Washa OR Cheraw OR Saura OR Cherokee OR Chickamauga OR Chiaha OR Chickahominy OR Chickanee OR Chiquini OR Chickasaw OR Chicora OR Chine OR Chisca OR Cisca OR Chitimacha OR Choctaw OR Houma OR Chowanoc OR Creek OR Congaree OR Canggaree OR Coree OR Coushatta OR Coharie OR Cusabo OR Eno people OR Garza OR Grigra OR Gris OR Guacata OR Santaluces OR Guacozo OR Guale OR Cusabo OR Iguaja OR Ybaja OR Guazoco OR Hitchiti OR "Hooks Nation" OR Chuaque OR Huaq OR Nuaq OR Jaega OR Jaupin OR Weapemoc OR Jobe OR Hobe OR JorORo OR Keyauwee OR Koasati OR Coushatta OR Koroa OR Luca people OR Lumbee OR Mabila OR Mobile OR Movila OR Machapunga OR Manahoac OR Mattaponi OR Matecumbe OR Matacumbses OR Matacumbe OR Matacombe OR "Mayaca people" OR Mayaimi OR Mayami OR Mayajuaca OR Meherrin OR Mikasuki OR Miccosukee OR Mocoso OR Monetons OR Monyton) | **77** |
| OR #13 | TITLE-ABS-KEY (Monekot OR Moheton OR Mougoulacha OR Muscogee OR Nahyssan OR Naniaba OR Nansemond OR Natchez OR Neusiok OR Newasiwac OR "Neuse River Indians" OR Nottaway OR Occaneechi OR Siouan OR Oconee people OR Ofo OR Okchai OR Ogchay OR Okelousa OR Opelousas OR Osochee OR Oswichee OR Usachi OR Oosecha OR Pacara OR Pakana OR Pacani OR Pagna OR Pasquenan OR “Pak-ka-na” OR Pacanas OR Pamlico OR Pamunkey OR Pascagoula OR Patiri OR “Pee Dee” OR Pedee OR Pensacola OR Potoskeet OR Quinipissa OR "Rappahannock Tribe" OR Saluda OR Saludee OR Saruti OR Santee OR Seretee OR Sarati OR Sati OR Sattees OR "Santa Luces" OR Saponi OR Saura OR Sawokli OR Sawakola OR Sabacola OR Sabacola OR Savacola OR Saxapahaw OR Sissipahua OR Shacioes OR Seminole OR Sewee OR Suye OR Joye OR Xoye OR Soya OR Shakori OR Shoccoree OR Stegarake OR Stuckanox OR Stukanox OR Sugeree OR Sagarees OR Sugaws OR Sugar OR Succa OR Surruque OR Suteree OR Sitteree OR Sutarees OR Sataree OR Taensa OR Talapoosa OR Tawasa OR Tequesta OR Terocodame OR Codam OR Hieroquodame OR Oodame OR Perocodame OR Teroodame OR Timucua OR Acuera OR “Agua Fresca” OR “Aqua Dulce” OR Freshwater OR Arapaha OR Cascangue OR Icafui OR Icafi OR Mocama OR Tacatacuru OR "Northern Utina" OR Ocale OR Oconi OR Potano OR Saturiwa OR Tucururu OR Tucuru OR Yufera OR Yui OR Ibi OR Yustaga OR Tiou OR Tioux OR Tocaste OR Tocobaga OR Tohome OR Tomahitan OR Topachula OR Tukabatchee OR Tuscarora OR Tuskegee OR Tutelo OR Tunica OR Tonica OR Tonnica OR Thonnica OR Uzita OR Vicela OR Viscaynos OR Waccamaw OR Wateree OR Guatari OR Watterees OR Waxhaw OR Waxsaws OR Wisack OR Wisacky OR Weesock OR Flathead OR Westo OR Winyaw OR Woccon OR Yamasee OR Yazoo OR Yuchi OR Euchee) | **94** |
| OR #14 | TITLE-ABS-KEY ("Ak Chin" OR "Southern Athabaskan" OR "Chiricahua Apache" OR "Jicarilla Apache" OR "Lipan Apache" OR "Mescalero Apache" OR Navajo OR Navaho OR Dine OR "San Carlos Apache" OR "Tonto Apache" OR Western OR "Coyotero Apache" OR "White Mountain Apache" OR Aranama OR Hanama OR Haname OR Chaimame OR Chariname OR Xaraname OR Taraname OR Coahuiltecan OR Cocopa OR Comecrudo OR Cotoname OR Genizaro OR Halchidhoma OR Hualapai OR Havasupai OR Hohokam OR Karankawa OR Kavelchadhom OR "La Junta" OR Mamulique OR Manso OR Maricopa OR Mojave OR Pima OR "Pima Bajo" OR "Pueblo people" OR "Ancestral Pueblo" OR Hano OR Hopi OR "Keres people" OR Acoma OR Cochiti OR Laguna OR "San Felipe" OR "Santa Ana" OR "Santo Domingo" OR Pueblo OR Zia OR Nambe OR Piro OR Pojoaque OR "San Ildefonso" OR Tesuque OR "Santa Clara" OR Isleta OR Picuris OR Sandia OR Taos OR "Ysleta del Sur" OR Tigua OR "Jemez Pueblo" OR Tewa OR "Ohkay Owingeh" OR Tiwa OR Towa OR "Zuni people" OR Quechan OR Yuma OR Quems OR Solano OR Tamique OR Toboso OR "Tohono Oodham" OR Qahatika OR Tompiro OR Ubate OR Walapai OR Yaqui OR Yoreme OR Yavapai OR Tolkapaya OR "Western Yavapai" OR Yavape OR "Northwestern Yavapai" OR Kwevkapaya OR "Southeastern Yavapai" OR Wipukpa OR "Northeastern Yavapai") | **1,540,068** |
| OR #15 | TITLE-ABS-KEY (Koori OR Koorie OR Ngunnawal OR Murri OR Murrdi OR Nyungar OR Yamatji OR Wangai OR Nunga OR Anangu OR Yapa OR Yolngu OR Bininj OR Tiwi OR Anindilyakwa OR Palawah OR Pallawah OR "Torres Strait Islander*" OR "Djabugay people") | **6,044** |
| OR #16 | TITLE-ABS-KEY (Fijian OR Papuans OR Wopkaimin OR Hewa OR Kaluli OR Dani OR "Ni-Vanuatu" OR Malaitan) | **4,904** |
|  | **#3 OR #4 OR #5 OR #6 OR #7 OR #8 OR #9 OR #10 OR #11 OR #12 OR #13 OR #14 OR #15 OR #16** | **2,121,507** |
|  | **#1 AND #2 AND (#3 OR #4 OR #5 OR #6 OR #7 OR #8 OR #9 OR #10 OR #11 OR #12 OR #13 OR #14 OR #15 OR #16)** | **8,280** |
| AND NOT #18 | animal* | **5,071** |
| Limited to articles published since 2000 | | **4,578** |
| Limited to articles in English | | **4,380** |

## Appendix III: Draft data extraction instrument

| **Assessment against eligibility criteria** | |
| --- | --- |
| Population |  |
| Concept |  |
| Context |  |
| Type of evidence source |  |
| **Evidence source details and characteristics** | |
| Citation details  (e.g. author(s), date, title, journal, volume, issue, pages) |  |
| Setting  (country/region/community) |  |
| Publication/research context  (based on needs identified by Indigenous community; part of broader tobacco elimination proposal; other) |  |
| Indigenous population(s) engaged, any sociodemographic descriptors  (age groups, gender, etc.) |  |
| **Details/results extracted from source of evidence** | |
| Any definition/characterization of tobacco resistance and/or elimination, and its strategies/goals/vision, by Indigenous populations engaged |  |
| Any narrative and/or quantitative assessment of effectiveness and impact  (considering cultural safety, acceptability, feasibility), specifically for Indigenous populations |  |
| Author recommendations regarding strategy implementation |  |
| Any challenges regarding implementation and possible solutions |  |
| Extent of Indigenous engagement from initiation through to implementation/evaluation | 1. Governance: |
|  | 1. Relationships: |
|  | 1. Prioritization: |
|  | 1. Methodologies: |
|  | 1. Participation: |
|  | 1. Capacity: |
|  | 1. Analysis and findings: |
|  | 1. Dissemination: |
