# Supplementary material for: Engagement of Indigenous peoples in commercial tobacco reform strategies: a scoping review protocol
Source: BMJ Open. 2025 Jul 3;15(7):e097952. doi: 10.1136/bmjopen-2024-097952 (PMC12226939; doi:10.1136/bmjopen-2024-097952)
Supplement: Supplementary file 2 [file bmjopen-2024-097952.reviewer_comments.pdf]

PEER REVIEW HISTORY

BMJ Open publishes all reviews undertaken for accepted manuscripts. Reviewers are asked to complete a checklist review form and are provided with free text boxes to elaborate on their assessment. These free text comments are reproduced below.

ARTICLE DETAILS

Title (Provisional)

Engagement of Indigenous peoples in commercial tobacco reform strategies: a scoping review protocol

Authors

Perusco, Andrew Daniel; Kennedy, Michelle; Tautolo, El-Shadan; Clark, Hershel; Henderson, Patricia Nez; Martinez, Sydney; Nez, Priscilla; Waa, Andrew; Wilcox, Sierra; Upton, Penney; Venugopal, Jeyasakthi; Maddox, Raglan

---

VERSION 1 - REVIEW

**Reviewer**	**1**
**Name**	**Andrew, Albert**
**Affiliation**	**The University of Auckland School of Medicine**
**Date**	**18-Feb-2025**
**COI**	**None**

Thank you for the opportunity to review the manuscript, tilted "Engagement of Indigenous peoples in commercial tobacco reform strategies: a scoping review protocol" in which the authors detail a research protocol for conducting a scoping review that help identify gaps and synthesise evidence on indigenous people experiences and engagement with tobacco resistance and eradication strategies.

Overall, this is a well-written study protocol on an important topic. However, I have a few suggestions that, if incorporated, could further enhance the quality of the paper

(1) The paper states that "reasons for exclusion at the full-text stage will be documented and reported in the final review." It would be helpful if the authors could briefly outline the potential reasons for article exclusion. They might consider presenting this information in a table format, listing inclusion and exclusion criteria side by side in separate columns.

(2) The search strategy string for the Scopus database has been provided. However, please clarify the search strings/keywords used for other databases; are they different? How were they adjusted? Consider including the full search strings for other databases for reference as appendix files.

(3) Will the same keywords used in the database searches also be applied when searching for relevant grey literature on databases that are not necessarily academic in nature, such as the Google search engine? If not, please explain why different keywords will be used.

(3) Please explain how disagreements in the data extraction process will be resolved.

(4) I could not find any information on how the authors will conduct the quality appraisal of the identified literature. Will any framework be used to evaluate the evidence from the selected/included studies? If so, what framework? If not, why not?

(5) I do wonder if it would be more beneficial if the authors discussed the strengths and limitations under a separate discussion heading instead of listing them below the abstract? Placing them in a dedicated discussion section could help improve clarity.

(6) Building on suggestion (5), the authors should also consider adding a paragraph (3-4 sentences) discussing the significance of the findings that could arise from this scoping review. How do they anticipate that the results of this scoping review will contribute to the discourse in this field or influence future health policy decisions? It is unclear from the current manuscript.

(7) For completeness, it may be helpful to include a brief timeline for project task completion. This could outline the anticipated dates for key milestones such as abstract screening, full-text screening, and data extraction and analysis, initial manuscript draft etc.

**Reviewer**	**2**
**Name**	**Carroll, Dana**
**Affiliation**	**University of Minnesota, psychiatry**
**Date**	**24-Mar-2025**
**COI**	**None**

Thank you for the opportunity to review this protocol for a scoping review. The goal of this review is to summarize the available evidence on commercial tobacco resistance and/or eradication strategies in relation to Indigenous peoples across Oceania, Pacific Islands and North America. Overall, this is an exceptionally well-written paper that has clearly outlined the proposed steps that the team will take to conduct the scoping review. I have one concern for addressing.

While the authors state that individual level interviews are outside the scoop of this review, it is not fully clear what is considered 'resistance and/or eradication strategies'. This comes to head when reviewing the table. Are these just example strategies? If so, can the title of the column be updated and this clarified in the text. For example, it was surprising to not see smokefree policies included. Also, what is meant by smoker's license? While there is listed 'redesign the cigarette to make it unappealing' does this include menthol flavor? This could

also include packaging or the cigarette itself? What about taxation to advantage cleaner forms (currently 'regulate' is included in this statement but not taxation). Also, what about health communications--media campaigns.. this is not an individual level intervention per se. Overall, i was confused by what did and did not get mentioned in this table. More clarity in these descriptions would be helpful.

Thank you for the opportunity to review.

VERSION 1 - AUTHOR RESPONSE

Feedback	Response	Location (within version with tracked edits)
Reviewer 1: Dr. Albert Andrew, The University of Auckland School of Medicine		
1. Thank you for the opportunity to review the manuscript, tilted "Engagement of Indigenous peoples in commercial tobacco reform strategies: a scoping review protocol" in which the authors detail a research protocol for conducting a scoping review that help identify gaps and synthesise evidence on indigenous people experiences and engagement with tobacco resistance and eradication strategies.<br>Overall, this is a well-written study protocol on an important topic. However, I have a few suggestions that, if incorporated, could further enhance the quality of the paper<br><br>The paper states that "reasons for exclusion at the full-text stage will be documented and reported in the final review." It would be helpful if the authors could briefly outline the potential reasons for article exclusion. They might consider	Thank you very much for taking the time to review our manuscript and for the valuable feedback.<br><br>We have now added a new table to the manuscript (Table 2) to summarise the inclusion criteria for the review, including possible reasons for exclusion.	Pages 6-7, Table 2

Feedback	Response	Location (within version with tracked edits)
presenting this information in a table format, listing inclusion and exclusion criteria side by side in separate columns.		
2. The search strategy string for the Scopus database has been provided. However, please clarify the search strings/keywords used for other databases; are they different? How were they adjusted? Consider including the full search strings for other databases for reference as appendix files.	We are still working with a health specialist librarian to finalise the search strategies for all databases. The search strategy piloted in Scopus, and as included in the supplementary file (Appendix I), will be adapted as appropriate to align with the syntax of each respective database. For example, there are different approaches to searching across fields such as the title, abstract and keywords of articles across databases. We will endeavour to make minimal modifications across these databases and the full search strategy will be submitted for publication along with the completed scoping review manuscript. As this is a protocol, we followed the guidance developed by members of the JBI Scoping Review Methodology Group, based on the PRISMA checklist for systematic review protocols: "Present a draft of the search strategy to be used for at least one electronic database, including planned limits, such that it could be repeated."[1]<br><br>1. Peters MDJ, Godfrey C, McInerney P, Khalil H, Larsen P, Marnie C, Pollock D, Tricco AC, Munn Z. Best practice guidance and reporting items for the development of scoping review protocols. JBI Evid Synth. 2022 Apr 1;20(4):953-968. https://doi.org/10.11124/jbies-21-00242	N/A
3. Will the same keywords used in the database searches also be applied when searching for relevant grey literature on databases that are not necessarily academic in nature, such as the Google search engine? If not,	After further consideration, we will now only be searching for relevant grey literature in ProQuest, given its advanced search function that would allow us to ensure consistent use of keywords and parameters across all databases. However, as we work through the review, we may conduct further searches in specific websites following consultation with	Page 7, Lines 10-12

Feedback	Response	Location (within version with tracked edits)
please explain why different keywords will be used.	the Indigenous Circle of the Society for Research on Nicotine and Tobacco. Any additional searches will be described in the final scoping review manuscript, including rationale for any modifications to the search strategy.	
4. Please explain how disagreements in the data extraction process will be resolved.	We have now added the following statement in the Methods:<br>"Any discrepancies in the information extracted from papers between the two reviewers will be resolved through full-text review and dialogue within the review team, including an Indigenous researcher(s) as required, in alignment with Indigenous data sovereignty principles."	Page 8, Lines 5-8
5. I could not find any information on how the authors will conduct the quality appraisal of the identified literature. Will any framework be used to evaluate the evidence from the selected/included studies? If so, what framework? If not, why not?	We will use the consolidated criteria for strengthening reporting of health research involving Indigenous peoples (CONSIDER checklist) [1] to appraise included papers. Each paper will be assessed against the eight research domains from the CONSIDER checklist: governance; relationships; prioritization; methodologies; participation; capacity; analysis and findings; and dissemination. We have now further clarified this in the Methods, under the *Data analysis and presentation* sub-heading.<br><br>We also note that critical appraisal of individual articles is not typical for scoping reviews as they are for systematic reviews:<br>"Typically, the goal of a scoping review is to address broader questions compared with a traditional systematic review. As such, the steps common to scoping reviews do not correspond exactly to those of a systematic review. Notably, critical appraisal (also called assessment of risk of bias or methodological quality assessment) of the sources of evidence is often omitted because the main purpose of a scoping review is to describe and map a body of literature in terms of characteristics and	Page 8, Lines 11-17

Feedback	Response	Location (within version with tracked edits)
	factors detailed by the review's objective, question/s, and inclusion criteria.” [2]<br><br>1. Huria T, Palmer SC, Pitama S, et al. Consolidated criteria for strengthening reporting of health research involving indigenous peoples: the CONSIDER statement. BMC Med Res Methodol. 2019;19: 173. https://doi.org/10.1186/s12874-019-0815-8<br>2. Peters MDJ, Godfrey C, McInerney P, Khalil H, Larsen P, Marnie C, Pollock D, Tricco AC, Munn Z. Best practice guidance and reporting items for the development of scoping review protocols. JBI Evid Synth. 2022 Apr 1;20(4):953-968. https://doi.org/10.11124/jbies-21-00242	
6. I do wonder if it would be more beneficial if the authors discussed the strengths and limitations under a separate discussion heading instead of listing them below the abstract? Placing them in a dedicated discussion section could help improve clarity.	We note that the section on *Strengths and Limitations of this Study* following the abstract is a requirement by the journal.<br><br>Although a Discussion section is not a requirement for protocol manuscripts, we have now added a brief *Expected Outcomes* paragraph with support from the journal editor: “The proposed scoping review will summarise published information on visions, goals and approaches to commercial tobacco eradication that supports the health, wellbeing and aspirations of Indigenous peoples, including any identified strengths, challenges and evaluations of proposed approaches. Findings may help contextualise the needs and priorities of Indigenous peoples as it relates to commercial tobacco, as well as necessary conditions and policies that would support the assertion of Indigenous sovereignty in implementing actions to prevent further harms. Importantly, this review is expected to help shift the field toward structural and system-level reform(s), rather than individualised health approaches. Findings will also directly support the work of the Tackling Indigenous Smoking (TIS) program across Australia and	Page 8, Lines 21-34

Feedback	Response	Location (within version with tracked edits)
	funded by the Australian Government of Health and Aged Care. The overarching goal of the TIS program is to improve the health and wellbeing of Aboriginal and Torres Strait Islander peoples through community-led, locally tailored actions geared towards eliminating and/or eradicating commercial tobacco and vaping related harms. This review will contribute to the development of Indigenous-led abolitionist strategies that aim to dismantle the structural conditions enabling the commercial tobacco epidemic."	
7. Building on suggestion (6), the authors should also consider adding a paragraph (3-4 sentences) discussing the significance of the findings that could arise from this scoping review. How do they anticipate that the results of this scoping review will contribute to the discourse in this field or influence future health policy decisions? It is unclear from the current manuscript.	Please see our previous response.	Page 8, Lines 21-34
8. For completeness, it may be helpful to include a brief timeline for project task completion. This could outline the anticipated dates for key milestones such as abstract screening, full-text screening, and data extraction and analysis, initial manuscript draft etc.	We appreciate that this would be helpful to include in the protocol manuscript. However, it is challenging to pinpoint certain timelines for each of the review stages as some stages would require more discussion as a group than others, and we are an international group of authors. Additionally, we are committed to publishing the protocol prior to progressing further with data collection for this scoping review, which adds further uncertainty around our timelines for completion. We will endeavour to include a brief timeline in our protocol's registration on Open Science Framework following the publication of this protocol: https://osf.io/wxqcb	N/A
Reviewer 2: Dr. Dana Carroll, University of Minnesota		

Feedback	Response	Location (within version with tracked edits)
1. Thank you for the opportunity to review this protocol for a scoping review. The goal of this review is to summarize the available evidence on commercial tobacco resistance and/or eradication strategies in relation to Indigenous peoples across Oceania, Pacific Islands and North America. Overall, this is an exceptionally well-written paper that has clearly outlined the proposed steps that the team will take to conduct the scoping review. I have one concern for addressing.<br>While the authors state that individual level interviews are outside the scoop of this review, it is not fully clear what is considered 'resistance and/or eradication strategies'. This comes to head when reviewing the table. Are these just example strategies? If so, can the title of the column be updated and this clarified in the text. For example, it was surprising to not see smokefree policies included. Also, what is meant by smoker's license? While there is listed 'redesign the cigarette to make it unappealing' does this include menthol flavor? This could also include packaging or the cigarette itself? What about taxation to advantage cleaner forms (currently 'regulate' is included in this statement but not taxation). Also, what about	Thank you very much for taking the time to review our manuscript and for the valuable feedback.<br><br>We have now clarified that Table 1 provides examples of tobacco endgame or eradication proposals, which were taken from a review conducted by McDaniel, Smith & Malone [1].<br><br>A smoker's license has been described by McDaniel, Smith & Malone [1] as follows: "Under a smoker licensing scheme those seeking to purchase tobacco from licensed retailers would obtain a 'smart swipecard' smoker's license, renewable annually, with purchase limits established by the user. Financial incentives to permanently relinquish the license could be offered, and new smokers seeking a license would demonstrate knowledge of tobacco's health and financial costs." A more detailed description of the proposed smoker's license is available in the cited review.<br><br>Based on the cited review, banning of menthol is captured under "Redeisgn the cigarette to make it unappealing." Packaging-related considerations are covered under "Regulated market model." We also confirm that taxation of combustible tobacco products is one of the regulatory tools discussed under "Regulate to advantage cleaner nicotine products over combustibles."<br>We agree with the reviewer that there are many more examples of commercial tobacco resistance and/or eradication strategies that have not been captured in Table 1. As noted earlier, Table 1 was intended to summarise examples of tobacco endgame or eradication proposals based on a previous review [1]. More detailed descriptions of each of the examples in Table 1 are discussed in-depth in the cited	Page 5, lines 10-16

Feedback	Response	Location (within version with tracked edits)
health communications--media campaigns.. this is not an individual level intervention per se. Overall, i was confused by what did and did not get mentioned in this table. More clarity in these descriptions would be helpful.	review. We also acknowledge that the absence of some examples reflects both the limitations of previous endgame frameworks and the need for Indigenous-led models of resistance that focus on systemic change, including commercial eradication and corporate accountability. These limitations are one reason we are undertaking this review—to (re)centre Indigenous worldviews in Indigenous commercial tobacco endgame strategy design and implementation. Strategies such as smokefree policies and tobacco-related health communications will be considered for inclusion.<br><br>1. McDaniel PA, Smith EA, Malone RE. The tobacco endgame: a qualitative review and synthesis. Tobacco Control. 2016;25(5):594-604. https://doi.org/10.1136/tobaccocontrol-2015-052356	
